# Early life stress explains reduced positive memory biases in remitted depression

**DOI:** 10.1016/j.eurpsy.2017.06.011

**Published:** 2017-09

**Authors:** J.A. Gethin, K.E. Lythe, C.I. Workman, A. Mayes, J. Moll, R. Zahn

**Affiliations:** a Division of Neuroscience and Experimental Psychology, The University of Manchester, Manchester M13 9PL, UK; b Cognitive and Behavioral Neuroscience Unit, D’Or Institute for Research and Education (IDOR), 22280-080 Rio de Janeiro, RJ, Brazil; c Institute of Psychiatry, Psychology & Neuroscience, Department of Psychological Medicine, Centre for Affective Disorders, King's College London, London SE5 8AZ, UK

**Keywords:** Major depressive disorder, Early life stress, Associative memory, Emotional bias, Overgeneralisation

## Abstract

**Background:**

There is contradictory evidence regarding negative memory biases in major depressive disorder (MDD) and whether these persist into remission, which would suggest their role as vulnerability traits rather than correlates of mood state. Early life stress (ELS), common in patients with psychiatric disorders, has independently been associated with memory biases, and confounds MDD versus control group comparisons. Furthermore, in most studies negative biases could have resulted from executive impairments rather than memory difficulties per se.

**Methods:**

To investigate whether memory biases are relevant to MDD vulnerability and how they are influenced by ELS, we developed an associative recognition memory task for temporo-spatial contexts of social actions with low executive demands, which were matched across conditions (self-blame, other-blame, self-praise, other-praise). We included fifty-three medication-free remitted MDD (25 with ELS, 28 without) and 24 healthy control (HC) participants without ELS.

**Results:**

Only MDD patients with ELS showed a reduced bias (accuracy/speed ratio) towards memory for positive vs. negative materials when compared with MDD without ELS and with HC participants; attenuated positive biases correlated with number of past major depressive episodes, but not current symptoms. There were no biases towards self-blaming or self-praising memories.

**Conclusions:**

This demonstrates that reduced positive biases in associative memory were specific to MDD patients with ELS rather than a general feature of MDD, and were associated with lifetime recurrence risk which may reflect a scarring effect. If replicated, our results would call for stratifying MDD patients by history of ELS when assessing and treating emotional memories.

## 1 Introduction

Patients with current major depressive disorder (MDD) have demonstrated biases towards better recall of negative than positive materials [1], [2], [3]. This is consistent with Bower's associative network theory of memory and emotion [4], which profoundly influenced the cognitive psychology of depression by proposing a close link between mood states, emotions, and memory. Despite nearly 40 years of researching this hypothesis, key questions remain. Two particularly relevant questions are whether emotional biases in memory can act as vulnerability factors outside of depressive episodes and whether they are specific for MDD.

The bias of better memory for negative compared to positive materials in MDD has been demonstrated most clearly with non-autobiographical stimuli and active recall tasks [5]. Using the Autobiographical Memory Test, however, impaired retrieval of contextual details for positive relative to negative events was only found in some studies in current [6] [7] and remitted MDD (rMDD) [8], [9]. A valence-independent impairment, however, was the most robust finding in a meta-analysis [10] and in the largest study in rMDD to date [11]. Despite inconsistencies about valence-effects, impaired autobiographical memory persisted into remission [8], [9], [11] suggesting its possible role in vulnerability [12]. Abnormalities on the Autobiographical Memory Test, however, are best accounted for on the basis of executive dyfunction [13], [14] rather than contextual memory per se, and performance is influenced by retrieval strategy [15].

Passive memory tasks avoid confounding patients’ performance with executive impairments, which could result simply from being distracted by depressive thoughts [16], although this literature is much sparser and more inconsistent. When priming is used to probe memory for semantically encoded materials, people with current MDD/dysthymia favour negative over positive materials, which is the opposite of healthy control participants [17]. In contrast, studies using a recognition memory task demonstrated intact recognition memory for both positive and negative materials [18], [19], [20] despite impaired valence-independent recall [19] in current MDD or, intriguingly, decreased negative and intact positive recognition memory in pregnant women with rMDD [21]. One study found subtle effects of personal relevance rather than valence or overall recognition memory performance in current MDD [20]. We found only one study demonstrating impaired emotional recognition memory in MDD, which was conducted in a symptomatic group and found no valence effect on accuracy [22]. One explanation for the heterogeneity in the passive memory literature that has not yet been investigated is that memory biases in MDD are due to the exposure of many patients to early life stress (ELS, [23]), which is usually absent in the typical healthy control population.

ELS itself was associated both with impaired retrieval of contextual details of emotional memories [24], [25], [26] and with a reduced positive memory bias [27]. Furthermore, ELS has been linked to stress hormone-induced medial temporal abnormalities in animals and humans [28]. The same medial temporal lobe structures have been demonstrated to underpin associative memory for temporal and spatial contexts in humans [29]. Some studies, however, report no consistent link between ELS and impaired emotional memory using the autobiographical memory test [30], [31] and a review suggested that experiencing depressive or post-traumatic reactions to stressors is necessary for impaired emotional memory, rather than stressful events or a history of ELS alone [32]. It is thus unclear whether MDD itself is associated with emotional memory biases, or whether this effect is mediated by ELS. This is because ELS has not been controlled for in the literature using more specific tests of contextual memory (i.e. passive memory tests) rather than those, which are confounded by executive functioning (e.g. Autobiographical Memory Test).

Given the close link between memories and mood postulated by Bower [4], one could postulate that the reduced positive memory biases sometimes reported in MDD contribute to the reductions in positive affect predicted to be specific to MDD by the decreased positive emotionality model of MDD [33]. In contrast, blame attribution models of MDD [34], [35] would predict that MDD vulnerability is related to selective overgeneralisation of self-blame-related memories, due to lack of access to contextual details, relative to blaming others (other-blame). This prediction would be made under the hypothesis that blame biases may be influenced by memory biases and vice versa. Corroborating evidence for a self-blaming emotional bias as a vulnerability factor for MDD was recently provided by showing reduced other-blaming relative to self-blaming emotions in rMDD [36], [37]. To our knowledge, self-blame-related memory biases have not been investigated in MDD, and the literature on the importance of self-reference effects when encoding emotional materials in mediating emotional memory biases in MDD is inconsistent [5], [17], [38].

In order to probe associative memory for temporal and spatial contexts of emotional materials per se, rather than the process of retrieving such information as probed on tasks, such as the Autobiographical Memory Test, we used a simple recognition memory task, which largely avoids the confounding effects of executive functions [39], [40]. This novel task was free of autobiographical components to allow strict experimental control of the relevant variables and is therefore only comparable with the Autobiographical Memory Test in that both require the spatio-temporal encoding and recognition of emotional information; otherwise these tests bear no resemblance. We designed this novel test through manipulation of temporal and spatial contextual details in statements describing social actions, derived from norms, which provided participants with positive and negative emotionally relevant concepts. This task was balanced across conditions to allow separate investigations of both valence- and blame-related biases. We investigated whether vulnerability to MDD rather than its symptoms is associated with emotional memory biases by studying a medication-free group of patients in full remission from symptoms [41], known to be at high lifetime risk of MDD [42], and compared against a healthy control group with no personal or family history of MDD. We probed whether MDD itself or only its interaction with ELS would be associated with emotional memory biases by comparing rMDD patients with and without a history of ELS.

We tested the alternative predictions of the self-blaming bias and positive emotionality models of vulnerability to MDD on associative memory for temporal and situational context. We favoured the hypothesis that rMDD patients would show self-blame-selective rather than negative or positive emotion-selective changes in associative memory compared to a healthy control (HC) group. We also hypothesised that this self-blaming bias would be stronger in patients with ELS. These hypotheses were based on our previous finding of an overall increase in proneness towards experimentally induced self-blame-related emotions (self-disgust/contempt) relative to blaming others (disgust/contempt towards others) in rMDD with no overall change in positive or negative emotional biases [36]. Given the proposed importance of the medial temporal lobe memory system in MDD [43] and its guilt-selective functional disconnection from the conceptual-semantic representations of social behaviour in the right anterior temporal lobe [44], we hypothesised that self-blaming emotional biases could arise in part by biasing associative memory mechanisms shown to be hosted by the medial temporal lobe [29].

## 2 Methods

### 2.1 Participants

Potential participants responded to print and online advertisements (see Table 1) for the UK Medical Research Council-funded project “Development of Cognitive and Imaging Biomarkers Predicting Risk of Self-Blaming Bias and Recurrence in Major Depression”. Suitable participants gave written informed consent and were assessed by a senior psychiatrist (RZ) and with the Structured Clinical Interview-I for DSM-IV-TR [45]. All participants were right handed as they also underwent neuroimaging. For inclusion in the rMDD group, participants had at least one previous MDE lasting at least two months, had been in remission for at least six months, and were free from centrally active medications (except hormonal contraceptives). They also had no current co-morbid or relevant past axis-I disorders to ensure group differences were due to vulnerability to MDD specifically rather than to the effects of other conditions. For the HC group, participants had no personal/first-degree family history of MDD. For full details of inclusion/exclusion criteria and recruitment procedures, see [36]. Participants were reimbursed for their time and travel costs. This research study was approved by the South Manchester NHS Research Ethics Committee (07/H1003/194).

**Table 1:** Reasons for exclusion of potential participants prior to memory task.

Reason for exclusion	*n*
*Following telephone screening interview*	
Current antihypertensive medications or statins	20
Current antidepressant or other centrally active medications	52
Diabetes	4
Epilepsy	5
Multiple sclerosis	3
Past cancer	7
Past stroke	1
Thyroid function problems	19
Vitamin D deficiency	1
Other psychiatric disorders than MDD	54
Substance or alcohol abuse	23
Other general medical condition	5
Family history of MDD/bipolar/schizophrenia (control group)	26
Excluded because of age-matching (control group)	3
Left-handed	20
MRI contraindications	77
Non-native English speaker	19
Out of age range	4
No reason recorded	5
Withdrawal after telephone screening interview	33
Not meeting full screening criteria for MDD	30
Had not been remitted from an episode for long enough	7
Fulfilled criteria for current MDD	13
*Total excluded after telephone screening interview*	431

*Following selection for initial assessment*	
Unable to schedule initial assessment	74
Fulfilled criteria for a bipolar disorder	6
Fulfilled criteria for current generalized anxiety disorder	1
Fulfilled criteria for current social anxiety disorder	7
MRI contraindications	1
Did not meet full criteria for MDD	5
Had not been remitted from an episode for long enough	3
Fulfilled criteria for past substance abuse	4
Probable personality disorder	2
Showed residual symptoms of post-traumatic stress disorder	3
Fulfilled criteria for current adjustment disorder	1
Fulfilled criteria for current MDD	1
Non-native English speaker	1
Fulfilled criteria for a past MDE that lasted for less than two months (control group)	1
Past depressive episode that did not fulfill criteria for past MDE (control group)	1
Probable or definite positive first degree family history of MDD (control group)	4
Withdrawal after the first assessment	1
Enrolled onto study prior to memory task development	38
Unable to schedule memory task session	27
Excluded because of age-matching for memory task (control group)	6
Ineligible for other tasks done in same session as memory task	4
*Total excluded from this session after selection for initial assessment*	191

In total, 707 participants consented to the telephone-screening interview. After exclusions, 85 participants (55 rMDD, 30 HC) completed the associative memory for social actions task. HC: healthy control; (r)MDD, (remitted) major depressive disorder; MDE: major depressive episode; MRI: magnetic resonance imaging.

In total, 707 participants gave oral consent to an initial telephone screening interview. Reasons for excluding participants are detailed in Table 1. Fifty-five rMDD and 30 HC participants completed the associative memory for social actions task. Data were excluded for two MDD participants due to current depression at the time of task completion and for six HC participants due to definite or questionable ELS. This paper reports a three-group comparison: rMDD with ELS (*n* = 25), rMDD without ELS (*n* = 28), and HC without ELS (*n* = 24). This was defined from a clinical interview as any of the following prior to the age of 18: separation from parents through death, divorce or adoption; threat of parental loss through near death; threatened or actual physical or sexual abuse; witnessing violence between/towards parents. Categorisation was conducted by two independent raters with high inter-rater reliability (*κ* = 0.947).

The three groups had comparable demographic characteristics: age (HC: median 27.5, range 20–64, rMDD without ELS: median 39.5, range 20–63, rMDD with ELS: median 34, range 18–64, *H* = 1.733, *P* = .420), years of education (HC: median 17.5, range 14–21.5, rMDD without ELS: median 17, range 12–21, rMDD with ELS: median 17, range 12–22, *H* = 2.602, *P* = .272), and gender (HC: 16 females, rMDD without ELS: 21 females, rMDD with ELS: 18 females, *χ*^2^ = .446, *P* = .8). There were no between-group differences in time between the encoding and retrieval stages of the memory task (*F*[2,74] = 1.407, *P* = .251).

All participants had Montgomery-Åsberg Depression Rating Scale [46] scores within the normal range and did not differ between groups (HC: median 0, range 0–2, rMDD without ELS: median 0, range 0–6, rMDD with ELS: median 0, range 0–4, *H* = 2.408, *P* = .3). Global Assessment of Functioning Scale [45] scores differed between the groups (HC: median 90, range 81–90, rMDD without ELS: median 90, range 70–90, rMDD with ELS: median 90, range 75–90, *H* = 11.998, *P* = .02). Follow-up Mann-Whitney tests showed no difference between the two rMDD groups (*U* = 307.5, *P* = .401). The HC group differed from both rMDD groups (with ELS: *U* = 167, *P* = .01, without ELS: *U* = 219, *P* = .04). All participants had no more than mild symptoms or functioning problems, however. Further clinical characteristics are detailed in Table 2.

**Table 2:** Clinical characteristics of the rMDD groups.

	rMDD without ELS (*n* *=* *28*)	rMDD with ELS (*n* *=* *25*)
Past MDD subtype		
With melancholic features	15/28	15/25
With atypical features	2/28	0/25
No specific subtype	11/28	10/25
Number of previous MDEs		
1	10	5
2	6	8
3	5	5
4	4	3
5	2	2
≥ 6	1	2
Average number of previous MDEs	2.46 ± 1.48 (range: 1–6)	4.44 ± 8.43 (range: 1–44)
Last MDE details		
Average length of MDE (months)	15.21 ± 13.74 (range: 0.5–60)	18.76 ± 23.60 (range: 1–96)
Average time in remission (months)	43.84 ± 56.27 (range: 6–282)	23.92 ± 15.76 (range: 6–60)
Severe MDE^a^	19/28	21/25
Moderate MDE^a^	9/28	4/25
Time without psychotropic medication (months)	46.79 ± 63.07 (range: 3–282)	52.57 ± 78.14 (range: 4–372)
Previous treatment		
SSRI	22/28	21/25
SNRI	0/28	1/25
Tricyclic antidepressant	3/28	1/25
Mirtazapine	1/28	1/25
Unknown class of antidepressant	4/28	3/25
Benzodiazepine	3/28	3/25
No antidepressant medication	4/28	2/25
CBT	8/28	7/25
Counselling	9/28	13/25
Self-guided CBT using internet or books	2/28	1/25
Previous suicide attempts	0.07 ± 0.26 (range: 0–1)	0.4 ± 1.15 (range: 0–5)
Lifetime axis-I co-morbidity^b^		
Bulimia nervosa	1/28	0/25
PTSD	2/28	0/25
No lifetime co-morbidity	25/28	25/25
First degree family history		
Relative with MDD	15/28	16/25
No relative with MDD, schizophrenia or bipolar disorder	11/28	9/25
Relative with schizophrenia	0/28	1/25
Relative with bipolar disorder	4/28	0/25

The Structured Clinical Interview-I for DSM-IV-TR was adapted to allow lifetime assessment of MDD subtypes, and showed excellent inter-rater reliability [36]. All participants had stopped medications well before the required washout phase. Participants with and without ELS did not differ on number of previous MDEs, average length of last MDE, average time in remission, average length since last use of psychotropic medications, and number of suicide attempts (*t* ≤ 1.710, *P* ≥ .093). Means and standard deviations (M ± SD) or number of cases are reported. CBT: cognitive behavioural therapy; ELS: early life stress; (r)MDD, (remitted) major depressive disorder, MDE: major depressive episode; PTSD: post-traumatic stress disorder; SSRI: selective serotonin reuptake inhibitor; SNRI: serotonin norepinephrine reuptake inhibitor.

a Based on ICD-10 criteria.

b All co-morbid disorders were fully remitted during the study and were not likely to be the primary cause of the MDEs.

### 2.2 Associative memory for social actions task

Before starting, participants were informed that they were completing a memory task, but not which particular aspect of the stimuli they would be tested on. Participants saw 80 written statements describing a specific social action occurring between themselves and their best friend. In each statement, the agent was either the participant (‘self-agency’, *n* = 40) or their best friend (‘other-agency’, *n* = 40). Statements in each agency condition were identical apart from the agency reversal and an irrelevant contextual detail (the time/place of the action). Statements in each agency condition were either positive (*n* = 20) or negative (*n* = 20), forming four conditions: self-praise, other-praise, self-blame and other-blame, e.g. “At your party, Paul spilled wine on your hall carpet” (other-blame; Paul is the best friend). Number of words was balanced between conditions. Stimuli were ordered randomly and presented for 6 s each. After reading each sentence, participants rated valence using a binary scale (good/bad). Task stimuli were developed from social scenarios generated by HCs [47] and a copy of the full task is available (http://www.translational-cognitive-neuroscience.org/start/test-materials).

Approximately 60 minutes after completing the task, participants were again presented with 80 stimuli, half of which were shown before and the rest were foils. Foils were identical to sentences shown previously but with a contextual detail changed. The change was irrelevant to the meaning of the social action, such as the time or place, e.g. “At your party, Paul spilled wine on your lounge carpet”. The number of foils was equal in each condition, as was the ratio of foils with a time- vs. place-related contextual change. Each stimulus appeared for 6 s; after each stimulus, participants had 3 s to make a forced choice on whether that exact sentence had appeared earlier (yes/no). Responses outside this time window (< 1% of all responses) were not recorded. One key for each response option was assigned to the index and middle fingers of the right hand (finger-to-response assignment was randomised across participants).

### 2.3 Data analysis

The proportion of hits and false alarms (adjusted by 0.5 trials in the appropriate direction if at ceiling/floor level [48]) were used to calculate *d’* scores; missed responses were removed (overall < 1% of trials were missed, and no more than four responses were missed by a participant in any one condition). The speed-accuracy trade-off score was calculated by dividing mean *d’* by mean response time (for missed responses, a maximum response time value of 3 s was assumed). This was based on the traditional measure of dividing mean response times by mean accuracy to capture speed and accuracy in a single performance measure [49]. These scores were then used to create three separate composite scores (self-blaming bias = self-minus other-blaming score, self-praising bias = self-minus other-praising score, positive bias = positive minus negative condition averages). An average score across conditions was also calculated.

All analyses were carried out in SPSS20 (http://www.spss.com). Data fulfilled the standard assumptions for each statistical test unless otherwise stated. To test our hypotheses, one-way ANOVAs for each composite score were used to investigate group differences at a two-sided alpha-level of *P* = .0125 (corresponding to an approximate Bonferroni-corrected *P* = *.*05 over the 4 composite scores we investigated) for the linear unweighted *F*-tests (to show that sample sizes were not a reflection of relative importance of each subgroup in the population). We also explored whether bias scores differed significantly from zero in each group at *P* = .05, one-sided (one-sample *t*-test). Where appropriate, results were confirmed by repeating tests with outlying values replaced with the mean ± 2.58 standard deviations.

## 3 Results

There were no group differences on average performance across conditions (*F*[2,74] = .123, *P* = .727). There were also no self-blaming biases in any group (*t* ≤ .512, *P* ≥ .613) and no differences between groups (*F*[2,74] = .001, *P* = .982). Likewise, there were no self-praising biases in any group (*t* ≤ .849, *P* ≥ .404) and no differences between groups (*F*[2,74] = .043, *P* = .837).

**Fig. 1:**
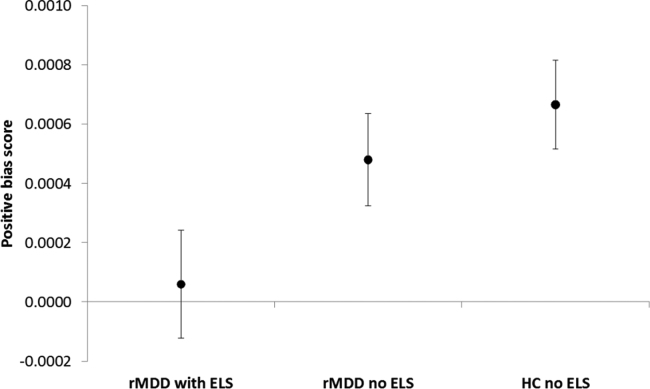
Means and standard errors of the means for positive bias scores (average positive score–average negative score) are displayed by group. The scores were calculated to reflect speed-accuracy trade-offs by dividing *d*’ scores by mean response times. Positive bias scores were significantly reduced in the rMDD with ELS group compared with the HC group, whereas rMDD without ELS did not differ from the HC group (see results for statistics). HC: healthy control; rMDD: remitted major depressive disorder; ELS: early life stress.

In contrast, both groups without a history of ELS showed positive biases (HC: *t*[23] = 4.450, *P* ≤ .0001; rMDD: *t*[27] = 3.095, *P* = .005) and these results were confirmed after outlier replacement (HC: unchanged; rMDD: *t*[27] = 3.232, *P* = .003), and after excluding 2 patients with remitted Post-traumatic Stress Disorder (PTSD: *t*[25] = 2.870, *P* = .008). The rMDD group with ELS, however, showed no bias for positive memories (*t*[24] = .324, *P* = .749). Positive bias scores differed between the three groups (*F*[2,74] = 6.592, *P* = .012, also after excluding 2 remitted PTSD patients: *F*[2,72] = 6.532, *P* = .013, Fig. 1). In post-hoc pairwise comparisons, the HC group showed a higher positive memory bias than the rMDD group with ELS (*P* = .012, Cohen's *d* = .74, Bonferroni-corrected *P* = .037) that was not driven by outliers (*P* = .012 in a separate two-sample *t*-test). There was no difference between HC and rMDD participants without ELS (*P* = .420, Cohen's *d* = .24, remaining after exclusion of 2 PTSD patients: *P* = 0.401) and a trend towards rMDD without ELS showing a higher positive bias compared with rMDD with ELS (*P* = .069, Cohen's *d* = .48).

The positive bias score in rMDD did not correlate with Beck Depression Inventory scores [50] (rho = –.157, *P* = .157), but correlated negatively with the number of past MDEs rho = –.271, *P* = .050, which strengthened after excluding an outlier on number of episodes: (rho = –.340, *P* = .014); this means that as the number of MDEs increased, the positive bias reduced. The positive valence bias score did not correlate with measures of executive function: verbal fluency (FAS score [51]; rho = –.041, *P* = .714) and set-shifting (trail-making test B-A [51], [52]; rho = .105, *P* = .347). The positive bias group differences were not present when analysing response time (*F*[2,74] < .0001, *P* *=* .983) or accuracy (*d*’ scores: *F*[2,74] = .818, *P* = .369) separately, thus only being present when using the *d*’/response time ratio capturing the speed/accuracy trade-off. Further supporting analyses demonstrated that group differences emerged only for the difference score which compares *d*’/response time ratios between the positive and negative conditions, but not for the *d*’/response time ratio in the positive (*F*[2,74] = .388, *P* = .680) and negative conditions separately (*F*[2,74] = 1.156, *P* = .320).

## 4 Discussion

This study investigated alternative predictions of blame attribution and emotional bias models for associative memory biases in people vulnerable to MDD in relation to ELS. In contrast to previous work, we employed a novel test that probes associative contextual memory for emotionally relevant materials without heavily confounding performance with executive demands. The results refuted our main hypothesis that rMDD patients, particularly those with ELS, show reduced contextual memory for self-blame-related stimuli compared with the HC group. Instead, rMDD patients with ELS exhibited a reduced positive memory bias compared with HC participants. In contrast, both HC and rMDD participants without ELS showed comparable positive memory biases. These results shed new light on contradictory findings of reduced positive memory biases reported in some studies in rMDD [8], [9] but not others [11]. Specifically, our study shows that controlling for ELS is crucial when investigating associative memory biases in MDD and the lack of control for ELS in previous studies may explain the variability in results.

Our findings are consistent with a previous study in women with past childhood sexual abuse and rMDD who displayed poorer retrieval of specific positive vs. negative memories [27]. Our findings extend these results through generalisation to males and showing that reduced positive memory biases in this group can be reproduced with a recognition memory task.

Conversely, an evaluative review [32] found ELS was not the primary factor in impairments on the Autobiographical Memory Test, but a PTSD/MDD diagnosis was more relevant, although valence biases were not evaluated. Our results confirm an interaction of MDD and ELS in their relationship with reduced positive memory biases as demonstrated by their dependence on both ELS and number of previous episodes. Our results further demonstrate that vulnerability to MDD itself is not associated with emotional memory abnormalities as rMDD without ELS showed normal positive memory biases.

Importantly, unlike the commonly used Autobiographical Memory Test [14], our positive memory bias score did not correlate with measures of executive function suggesting it is a true measure of impaired access to/encoding of associative memories rather than of general executive difficulties. In this study, we defined ELS as a fixed set of potentially stressful events in childhood, rather than by the response of the participant to that stressor. It has been suggested [32] that a traumatic response to ELS drives emotional memory difficulties, rather than experiencing a stressful event per se. Although the severity of response to each stressor was not assessed, all our participants were screened for PTSD history. Two participants met PTSD criteria in adulthood, which was remitted at the time of study entry. Analyses repeated after removing these participants confirmed the results, thereby showing that PTSD did not drive any effects. Therefore, this study shows that ELS even in the absence of PTSD can result in emotional memory changes.

On a more cautionary note, one limitation of our study was that we were unable to include a neutral comparison condition and therefore only assessed differences between the positive and negative conditions, which we referred to as positive/negative biases. One might argue that we were unable to disentangle the respective contribution of positive and negative memories separately. Both better memory for negative, as well as poorer memory for positive materials could have contributed to the abnormal results in MDD patients with ELS compared with the other groups. One further caveat is that the reduction in positive memory biases in patients with MDD and ELS could only be detected when combining measures of speed and accuracy such that it is difficult to compare with the previous literature, which has usually examined these separately.

In summary, our findings demonstrate a loss of positive bias for associative memory in rMDD patients with a history of ELS. This was in contrast to the comparable degrees of positive biases observed in the rMDD and HC groups without ELS. Positive biases decreased with an increasing number of MDEs suggesting MDEs may re-activate traumatic memories, thereby increasing vulnerability by further decreasing positive memory biases which could contribute to the postulated scarring effects of MDEs [53]. Increased focus on positive affect in depression therapy, including a focus on positive memory recall, has recently been suggested [54], particularly when these memories are concordant with one's current view of self [55]. If replicated, our results would call for stratifying patients according to ELS in future clinical trials to investigate differential treatment effects. Further, we demonstrated that self-blaming emotional biases previously shown in our MDD group [36] did not arise at the level of associative contextual memory. This is intriguing as it suggests that self-blaming emotional biases in MDD arise at the semantic level [47] rather than at the level of associative memory for temporal and spatial context. This hypothesis could be investigated more directly in future studies on the formation of context-independent (i.e. semantic) emotional memories in patients with MDD.

## Funding and other support

This work was funded by an MRC clinician scientist fellowship (G0902304) to Roland Zahn. Jennifer A. Gethin was funded by an EPSRC PhD studentship.

## Disclosure of interest

The authors declare that they have no competing interest.

## References

[1] Dalgleish T., Werner-Seidler A. (2014). Disruptions in autobiographical memory processing in depression and the emergence of memory therapeutics. Trends Cogn Sci.

[2] Gotlib I.H., Joormann J., NolenHoeksema S., Cannon T.D., Widiger T. (2016). Cognition and depression: current status and future directions.

[3] Elliott R., Zahn R., Deakin J.F., Anderson I.M. (2011). Affective cognition and its disruption in mood disorders. Neuropsychopharmacology.

[4] Bower G.H. (1981). Mood and memory. Am Psychol.

[5] Matt G.E., Vazquez C., Campbell W.K. (1992). Mood-congruent recall of affectively toned stimuli – a meta-analytic review. Clin Psychol Rev.

[6] Williams J.M.G., Scott J. (1988). Autobiographical memory in depression. Psychol Med.

[7] Kaviani H., Rahimi-Darabad P., Naghavi H.R. (2005). Autobiographical memory retrieval and problem-solving deficits of Iranian depressed patients attempting suicide. J Psychopathol Behav Assess.

[8] Park R.J., Goodyer I.M., Teasdale J.D. (2002). Categoric overgeneral autobiographical memory in adolescents with major depressive disorder. Psychol Med.

[9] Gupta R., Kar B.R. (2012). Attention and memory biases as stable abnormalities among currently depressed and currently remitted individuals with unipolar depression. Front Psychiatry.

[10] Liu X., Li L., Xiao J., Yang J., Jiang X. (2013). Abnormalities of autobiographical memory of patients with depressive disorders: a meta-analysis. Psychol Psychother.

[11] Spinhoven P., Bockting C.L.H., Schene A.H., Koeter M.W.J., Wekking E.M., Williams J.M.G. (2006). Autobiographical memory in the euthymic phase of recurrent depression. J Abnorm Psychol.

[12] van Minnen A., Wessel I., Verhaak C., Smeenk J. (2005). The relationship between autobiographical memory specificity and depressed mood following a stressful life event: a prospective study. Br J Clin Psychol.

[13] Williams J.M., Barnhofer T., Crane C., Herman D., Raes F., Watkins E. (2007). Autobiographical memory specificity and emotional disorder. Psychol Bull.

[14] Dalgleish T., Williams J.M., Golden A.M., Perkins N., Barrett L.F., Barnard P.J. (2007). Reduced specificity of autobiographical memory and depression: the role of executive control. J Exp Psychol.

[15] Griffith J.W., Sumner J.A., Debeer E., Raes F., Hermans D., Mineka S. (2009). An item response theory/confirmatory factor analysis of the Autobiographical Memory Test. Memory.

[16] Watts F.N., Dalgleish T., Bourke P., Healy D. (1990). Memory deficit in clinical depression: processing resources and the structure of materials. Psychol Med.

[17] Watkins P.C. (2002). Implicit memory bias in depression. Cogn Emot.

[18] Olsen E.K., Bjorkquist O.A., Bodapati A.S., Shankman S.A., Herbener E.S. (2015). Associations between trait anhedonia and emotional memory deficits in females with schizophrenia versus major depression. Psychiatry Res.

[19] Liu W.H., Wang L.Z., Zhao S.H., Ning Y.P., Chan R.C.K. (2012). Anhedonia and emotional word memory in patients with depression. Psychiatry Res.

[20] Wittekind C.E., Terfehr K., Otte C., Jelinek L., Hinkelmann K., Moritz S. (2014). Mood-congruent memory in depression – The influence of personal relevance and emotional context. Psychiatry Res.

[21] Williams M.E., Becker S., McKinnon M.C., Wong Q., Cudney L.E., Steiner M. (2015). Emotional memory in pregnant women at risk for postpartum depression. Psychiatry Res.

[22] Yeh Z.T., Hua M.S. (2009). Effects of depressive disorder on false memory for emotional information. Depress Anxiety.

[23] Chapman D.P., Whitfield C.L., Felitti V.J., Dube S.R., Edwards V.J., Anda R.F. (2004). Adverse childhood experiences and the risk of depressive disorders in adulthood. J Affect Disord.

[24] Crane C., Heron J., Gunnell D., Lewis G., Evans J., Williams J.M.G. (2014). Childhood traumatic events and adolescent overgeneral autobiographical memory: findings in a UK cohort. J Behavr Ther Exp Psychiatry.

[25] Hitchcock C., Nixon R.D.V., Weber N. (2014). A review of overgeneral memory in child psychopathology. Br J Clin Psychol.

[26] Burnside E., Startup M., Byatt M., Rollinson L., Hill J. (2004). The role of overgeneral autobiographical memory in the development of adult depression following childhood trauma. Br J Clin Psychol.

[27] Aglan A., Williams J.M.G., Pickles A., Hill J. (2010). Overgeneral autobiographical memory in women: association with childhood abuse and history of depression in a community sample. Br J Clin Psychol.

[28] McEwen B.S., Nasca C., Gray J.D. (2016). Stress effects on neuronal structure: hippocampus, amygdala, and prefrontal cortex. Neuropsychopharmacology.

[29] Mayes A., Montaldi D., Migo E. (2007). Associative memory and the medial temporal lobes. Trends Cogn Sci.

[30] Wessel I., Meeren M., Peeters F., Arntz A., Merckelbach H. (2001). Correlates of autobiographical memory specificity: the role of depression, anxiety and childhood trauma. Behav Res Ther.

[31] Peeters F., Wessel I., Merckelbach H., Boon-Vermeeren M. (2002). Autobiographical memory specificity and the course of major depressive disorder. Comprehensive Psychiatry.

[32] Moore S.A., Zoellner L.A. (2007). Overgeneral autobiographical memory and traumatic events: an evaluative review. Psychol Bull.

[33] Watson D., Clark L.A., Carey G. (1988). Positive and negative affectivity and their relation to anxiety and depressive disorders. J Abnorm Psychol.

[34] Abramson L.Y., Seligman M.E.P., Teasdale J.D. (1978). Learned helplessness in humans – critique and reformulation. J Abnorm Psychol.

[35] Kinderman P., Bentall R.P. (1997). Causal attributions in paranoia and depression: internal, personal, and situational attributions for negative events. J Abnorm Psychol.

[36] Zahn R., Lythe K.E., Gethin J.A., Green S., Deakin J.F., Workman C. (2015). Negative emotions towards others are diminished in remitted major depression. Eur Psychiatry.

[37] Green S., Moll J., Deakin J.F., Hulleman J., Zahn R. (2013). Proneness to decreased negative emotions in major depressive disorder when blaming others rather than oneself. Psychopathology.

[38] Blaney P.H. (1986). Affect and memory – a review. Psychol Bull.

[39] Kopelman M.D., Stanhope N. (1998). Recall and recognition memory in patients with focal frontal, temporal lobe and diencephalic lesions. Neuropsychologia.

[40] Haist F., Shimamura A.P., Squire L.R. (1992). On the relationship between recall and recognition memory. J Exp Psychol.

[41] Bhagwagar Z., Cowen P.J. (2008). ‘It's not over when it's over’: persistent neurobiological abnormalities in recovered depressed patients. Psychol Med.

[42] Eaton W.W., Shao H., Nestadt G., Lee H.B., Bienvenu O.J., Zandi P. (2008). Population-based study of first onset and chronicity in major depressive disorder. Arch Gen Psychiatry.

[43] Clark L., Chamberlain S.R., Sahakian B.J. (2009). Neurocognitive mechanisms in depression: implications for treatment. Ann Rev Neurosci.

[44] Green S., Ralph M.A.L., Moll J., Deakin J.F.W., Zahn R. (2012). Guilt-selective functional disconnection of anterior temporal and subgenual cortices in major depressive disorder. Arch Gen Psychiatry.

[45] First M.B., Spitzer R.L., Gibbon M., Williams J.B.W. (2002). Structured Clinical Interview for DSM-IV-TR Axis I Disorders, Research Version, Patient Edition. (SCID-I/P).

[46] Montgomery S.A., Åsberg M. (1979). A new depression scale designed to be sensitive to change. Br J Psychiatry.

[47] Green S., Lambon Ralph M.A., Moll J., Zakrzewski J., Deakin J.F.W., Grafman J. (2013). The neural basis of conceptual – emotional integration and its role in major depressive disorder. Soc Neurosci.

[48] Durrant S.J., Cairney S.A., Lewis P.A. (2013). Overnight consolidation aids the transfer of statistical knowledge from the medial temporal lobe to the striatum. Cerebral Cortex.

[49] Townsend J.T., Ashby F.G. (1983). The stochastic modeling of elementary psychological processes.

[50] Beck A.T., Steer R.A., Garbin M.G. (1988). Psychometric properties of the Beck Depression Inventory – 25 years of evaluation. Clin Psychol Rev.

[51] Spreen O., Strauss E. (1998). A compendium of neuropsychological tests: administration, norms, and commentary.

[52] Saraswat N., Ranjan S., Ram D. (2006). Set-shifting and selective attentional impairment in alcoholism and its relation with drinking variables. Indian J Psychiatry.

[53] Post R.M. (1992). Transduction of psychosocial stress into the neurobiology of recurrent affective disorder. Am J Psychiatry.

[54] Dunn B.D. (2012). Helping depressed clients reconnect to positive emotion experience: current insights and future directions. Clin Psychol Psychother.

[55] Werner-Seidler A, Tan L, Dalgleish T. The vicissitudes of positive autobiographical recollection as an emotion regulation strategy in depression. Clin Psychol Sci 2017. doi:2167702616647922.

